# Cross-Sectional Analysis of Hypoxia-Regulated *miRNA-181a*, *miRNA-199a*, HIF-1α, and SIRT1 in the Development of Type 2 Diabetes in Patients with Obstructive Sleep Apnea—Preliminary Study

**DOI:** 10.3390/jcm13247644

**Published:** 2024-12-15

**Authors:** Filip Franciszek Karuga, Piotr Kaczmarski, Marcin Sochal, Bartosz Szmyd, Piotr Białasiewicz, Agata Gabryelska

**Affiliations:** 1 Department of Sleep Medicine and Metabolic Disorders, Medical University of Lodz, 90-419 Lodz, Poland; piotr.kaczmarski@umed.lodz.pl (P.K.); marcin.sochal@umed.lodz.pl (M.S.); piotr.bialasiewicz@umed.lodz.pl (P.B.); 2 Department of Neurosurgery and Neuro-Oncology, Barlicki University Hospital, Medical University of Lodz, 90-419 Lodz, Poland; bartosz.szmyd@umed.lodz.pl; 3 Department of Pediatrics, Oncology, and Hematology, Medical University of Lodz, 90-419 Lodz, Poland

**Keywords:** hypoxia, diabetes mellitus, obstructive sleep apnea, microRNA-181a, microRNA-199a, sirtuin1, SIRT1

## Abstract

**Introduction**: Obstructive sleep apnea (OSA) is recognized as an independent risk factor for diabetes mellitus type 2 (T2DM) development, which is twice as common in patients with OSA compared to non-OSA patients. **Objectives**: This study aimed to investigate changes in oxygen metabolism and their role in T2DM development among OSA patients through epigenetic processes via *miRNA-181a*, *miRNA-199a*, and enzymatic processes via SIRT1 and HIF-1α. **Methods**: Based on polysomnography, apnea–hypopnea index and the presence of T2DM patients were divided into three groups: control group (*n* = 17), OSA group (*n* = 11), OSA&T2DM (*n* = 20) group. Total RNA was extracted from the buffy coat. Moreover, HOMA-IR (Homeostatic Model Assessment for Insulin Resistance) was counted. **Results**: Morning *miRNA-181a* expression was significantly higher in the OSA&T2DM group than in the control group: 67.618 vs. 32.685 (*p* = 0.036). Evening *miRNA-199a* expression was significantly higher in the OSA group than in the control group: 5.043 vs. 2.081 (*p* = 0.042), while its morning expression was significantly higher in the OSA&T2DM group when compared to the control: 4.065 vs. 1.605 (*p* = 0.036). *MiRNA-181a* evening expression revealed a negative correlation with the SIRT1 evening and morning expressions (R = −0.367, *p* = 0.010 and R = −0.405, *p* = 0.004, respectively). Moreover, morning *miRNA-181a* was positively correlated with HOMA-IR (R = 0.321, *p* = 0.034). *MiRNA-199a* evening expression presented a moderate positive correlation with the *SIRT1* morning expressions (R = 0.48, *p* < 0.001) and HOMA-IR (R = 0.35, *p* = 0.02). **Conclusions**: Patients suffering from OSA and T2DM had an increased expression of *miRNA-181a*. Moreover, a negative correlation between *miRNA-181a* and *SIRT1* expression was observed, while a correlation between *miRNA-181a* and insulin resistance was positive. This phenomenon might suggest a possible epigenetic pathway for an increased incidence of T2DM in OSA patients however further research is needed.

## 1. Introduction

Obstructive sleep apnea (OSA) is a chronic condition characterized by recurrent pauses in breathing during sleep, which leads to intermittent hypoxia (IH), hypercapnia, arousals, and sleep fragmentation. Recent data suggests that severe and moderate forms of OSA affect between 6 and 17% of adults in the general population, while other research suggests that the prevalence of OSA may reach up to 23% among women and 49% among men. It is estimated that the male to female ratio is between 3:1 and 5:1. The gold standard in OSA diagnosis is a nocturnal polysomnography examination (PSG). Based on the number of apneas and hypopneas per hour of sleep, the apnea–hypopnea index (AHI) is calculated. AHI defines the severity of OSA as 15 > AHI ≥ 5–mild, 30 > AHI ≥ 15–moderate and AHI ≥ 30–severe. Decreased sleep quality caused by OSA results in excessive daytime sleepiness, cognitive impairment, and an increased risk of car accidents. Additionally, OSA is recognized as an independent risk factor for cardiovascular (CVD) and metabolic diseases, including diabetes mellitus type 2 (T2DM) [1,2,3,4,5]. In the study of Mahmood et al., it was found that the prevalence of T2DM in OSA patients was 30.1% while in the group without OSA, the prevalence is only 18.6% [6]. The coexistence of these two diseases is the subject of numerous scientific papers, which investigated many possible mechanisms of that interaction [7]. Nevertheless, the association is still not fully understood. The most effective form of treatment for OSA is continuous positive air pressure (CPAP) therapy, which generates constant air pressure in the upper airways preventing their collapse and eliminating the recurrent periods of hypoxia. Unfortunately, CPAP treatment seems to be ineffective in glycemic control in patients with already developed T2DM however it can possibly slow down the onset of T2DM [8].

The main factor responsible for oxygen metabolism homeostasis is hypoxia-inducible factor (HIF). HIF is a heterodimer composed of two subunits: α-subunit (HIF α), which is oxygen-regulated, and constitutively expressed β-subunit (HIF β). Subunit α is oxygen sensitive. The three analogs of HIF α-subunits are known as the following: HIF-1α, HIF-2α, and HIF-3α. HIF-1α is an important transcription factor responsible for the activation of over 1000 different genes thereby affecting metabolic processes, angiogenesis, erythropoiesis, and others [9]. It has been shown that HIF-1α is upregulated in OSA patients [10]. Its role in T2DM development in OSA patients has been already extensively studied. 

Sirtuin 1 (SIRT1) is a nicotinamide adenosine dinucleotide (NAD)-dependent deacetylase that removes acetyl groups from a variety of proteins. SIRT1 belongs to the sirtuin family, which is composed of seven members. SIRT1 is the most widely studied due to its significance in the mechanisms regulating various physiological and pathological processes, including apoptosis and inflammation [11]. Over the last few years, the regulation of HIF-1α by sirtuin family members has been a topic of extensive interest and a subject of controversy [12]. Dioum et al. discovered that the sirtuin family regulates hypoxic signaling with diverse redox-sensitive mechanisms. In this study, the aforementioned impact was found only in the case of HIF-2α, but not HIF-1α [13]. Lim et al. found that SIRT1 inactivated HIF-1α signaling by deacetylation at the Lys674 of HIF-1α, followed by blocking transcriptional coactivator p300 recruitment [14]. Further studies showed that SIRT1 leads to HIF-1α stabilization via direct binding and deacetylation in a hypoxic environment. This conclusion was based on the inactivation or depletion of SIRT1 leading to an impaired accumulation of HIF-1α protein during hypoxia accompanied by heightened acetylation [12,15]. SIRT1 levels in blood were found to be decreased in the OSA patients when compared with the control patients. Additionally, 3-month CPAP treatment restored SIRT1 blood levels and its activity [16]. SIRT1 is an important regulatory factor in metabolic disorders including T2DM [17,18,19]. Studies of T2DM in mouse models showed an increased expression of protein tyrosine phosphatase 1B (PTB1B), which is an inhibitor of the insulin receptors and leads to insulin resistance. SIRT1 inhibits PTB1B and sensitizes cells to insulin [20]. So decreased SIRT1 levels in the cells of the OSA patients can lead to the development of T2DM. Moreover, in another study on transgenic mice models, it was shown that SIRT1 increases insulin secretion in response to KCL and glucose [21]. SIRT1 has gluconeogenic activity by inducing the overexpression of genes of the hepatic pathway through peroxisome proliferator-activated receptor gamma coactivator 1-alpha during fasting [22]. This evidence shows that the decreased levels of SIRT1 in the OSA patients can disrupt glucose metabolism.

MicroRNAs (miRNAs) are small, 18–24 nucleotide, nonprotein coding RNA molecules that regulate eukaryotic gene expression by binding to target mRNA, which leads to either degradation or translational repression. They play a crucial role in a variety of biological processes, such as apoptosis, stress response, proliferation, and metabolism [23,24]. Additionally, several studies have determined differently expressed miRNAs in response to hypoxia, also in the OSA patients [25,26]. Many of the present miRNAs altered expression levels in response to hypoxia, which depends on the duration of exposition and oxygen concentration. In the study of Li et al., a total number of 104 differently expressed miRNAs in the serum of the OSA patients and non-OSA controls were identified. Ninety-four of the miRNAs were downregulated and ten were upregulated. A high degree of differential expression was determined for *miRNA485-5p*, *miRNA107*, *miRNA574-5p*, *miRNA-199-3p*, *miRNA127-3p*, and *miRNA139-3p*. However, the study had only 6 patients, 3 in each group [27]. Further studies by Santamaria-Martos et al. investigated 188 different miRNAs and revealed significant differences in 10 miRNAs expressions, including *miRNA199a* and *miRNA181a-2*, comparing the plasma of the OSA study group (21 patients) and the non-OSA controls (6 patients). Later, the results were validated with 139 OSA patients and 64 non-OSA controls. Finally, six validated miRNAs were selected: *miRNA181a*, *miRNA199-b*, *miRNA-345*, *miRNA133a*, *miRNA-340*, and *miRNA 486-3p*. After 6-month CPAP treatment in the same group of patients, a significant change in miRNAs levels was found only in the case of *miRNA345* [25]. Lacedonia et al. described another four miRNAs that were increased in the OSA patients compared to the non-OSA controls: *miRNA21*, *miRNA23b*, *miRNA145*, and *miRNA210* [28]. However, these results are not consistent with the study of Santamaria-Martos et al., which indicated *miRNA21* and *miRNA145* as possible endogenous controls, because they were only slightly decreased in the OSA patients [29].

**Figure 1:**
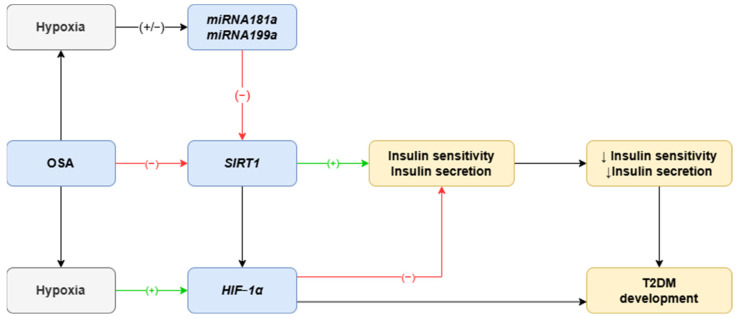
Role of *miRNA181a*, *miRNA199a*, *HIF-1α*, and *SIRT1* in development of T2DM in OSA patients. *SIRT1* increases insulin sensitivity and insulin secretion. Level of SIRT1 is lower in patients suffering from OSA, which is responsible for insulin resistance and insulin-reduced secretion followed by T2DM development. Hypoxia-sensitive *miRNAs* are one possible mechanisms of SIRT1 decreasing in OSA patients. Moreover, SIRT1 may act via HIF-1alfa, which is responsible for activation of vast number of genes. OSA—obstructive sleep apnea; T2DM—type 2 diabetes mellitus; SIRT1—sirtuin 1; HIF-1α—hypoxia-inducible factor 1α.

Due to financial constraints, this preliminary study focused on a limited selection of miRNAs for measurement. We established the following criteria for selection: responsiveness to hypoxia and targeting of SIRT1. Five miRNAs met these criteria: *miRNA-133*, *miRNA-181a*, *miRNA-199a*, *miRNA-485*, and *miRNA-486*. Among these, *miRNA-181a* and *miRNA-199a* were chosen based on their high hypoxia sensitivity, strong evidence supporting their reliability, and superior target scores for SIRT1 (see Figure 1) [25,27,30].

This study aimed to investigate the possible mechanisms involved in distinct changes in oxygen metabolism and their role in T2DM development among OSA patients through epigenetic (hypoxia-sensitive miRNAs: *miRNA-181a*, *miRNA-199a*) and enzymatic processes (SIRT1, HIF-1α).

## 2. Materials and Methods

This study was conducted in the Department of Sleep Medicine and Metabolic Disorders at the Medical University of Lodz from April 2018 until February 2024. Participants were recruited at the Sleep and Respiratory Disorders Centre (Central Clinical Hospital of the Medical University of Lodz). The study was approved by the Bioethical Committee of the Medical University of Lodz (Number of the Bioethical Committee Consent RNN/77/18/KE with an extension KE/1137/20, 13/03/2018). All patients underwent standard physical examination. The diagnosis of T2DM was made based on the patients’ medical documentation or the patients’ clinical factors (relying on the recommendations of the Polish Diabetes Society) [31]. Patients included in the study needed to meet the following criteria: written, informed patient’s consent to participate in the study, age between 30 and 70 years old, and BMI > 20 kg/m^2^ and <45 kg/m^2^. The following were the exclusion criteria: chronic pulmonary disease, history of infection 2 weeks prior and after the PSG examination, active or history of cancer, lifetime history diagnosed sleep disorders other than OSA, clinical depression or other psychiatric disorders, employment in changing shift system, intercontinental flight 2 weeks prior to PSG, abuse of or dependency on alcohol or illegal drugs, caffeine intake >900 mg per day, use of hypnotic medication or medication known to affect sleep during the 2 weeks before PSG.

The participants arrived at the sleep laboratory around 9 PM, with a possible variation of 30 min, and underwent a physical examination, which included measuring their body weight, height, heart rate, and blood pressure. During PSG, the following channels were recorded: electroencephalography (C4\A1, C3\A2), electromyography of the chin muscles and anterior tibialis, electrooculography, oronasal airflow (measured with a thermistor), snoring, body position, respiratory movements of the chest and abdomen (measured with piezoelectric sensors), unipolar electrocardiogram, and oxygen saturation (SpO_2_). The PSG was conducted using an Alice 6 device (Philips-Respironics, Murrysville, PA, USA). Sleep stages were scored based on the 30 s epoch standard. Apnea was defined as a reduction in airflow to less than 10% of baseline for at least 10 s. Hypopnea was characterized by a reduction in airflow of at least 30% for at least 10 s, accompanied by a decrease in SpO_2_ of over 3% or arousal. The collected data were exported to EDF files and then edited, processed, and analyzed using Neuro-Analyzer v0.23.9. Arousal scoring followed the guidelines of the American Academy of Sleep Medicine.

**Figure 2:**
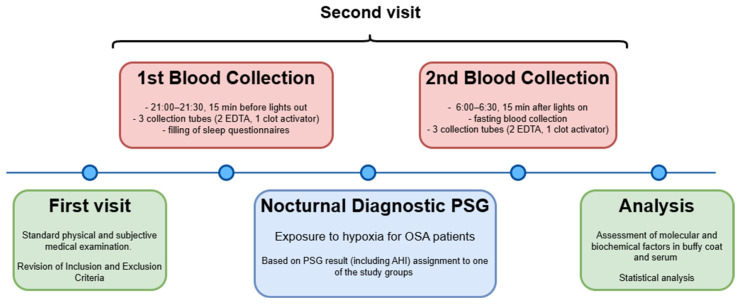
Timetable of study protocol. PSG—polysomnography, AHI—apnea–hypopnea index.

Blood samples were collected in the evening (at 21:00–21:30, 15 min before lights out), and in the morning (at 6:00–6:30, 15 min after lights on) following the PSG examination, and then centrifuged (see Figure 2). The serum and buffy coat were collected and stored at −80 °C. Fasting glucose and insulin levels in the serum from the morning blood were assessed. The insulin resistance was determined using the homeostasis model assessment-estimated insulin resistance (HOMA-IR) formula.

$$\text{HOMA-IR}=\frac{\left[\mathrm{glucose}\left(\frac{\mathrm{mg}}{\mathrm{dL}}\right)\right]\times \left[\mathrm{insulin}\left(\frac{\mathrm{mU}}{L}\right)\right]}{405}$$

In the next step, the following molecular and biochemical factors were assessed: *SIRT1* expression at mRNA level, *miRNA-181a* and *miRNA-199a* expression, SIRT1 intracellular protein levels through ELISA, and HIF-1α serum protein level. These specific miRNAs were chosen based on the literature; their capability to target SIRT1 was additionally validated using miRDB www.mirdb.org (accessed on 3 April 2021) [30].

Total RNA was extracted from the buffy coat using the mirVana PARIS Protein and RNA Isolation System (Invitrogen, Carlsbad, CA, USA) according to the manufacturer’s protocol. The quantity and quality of the RNA were measured with a PicoDrop spectrophotometer (Picodrop Limited, Hinxton, UK). The purified total RNA was immediately used for cDNA synthesis or stored at −80 °C. Reverse transcription was performed using a Maxima First Strand cDNA Synthesis Kit (Thermo Fisher Scientific, Waltham, MA, USA) according to the manufacturer’s recommendations. Expression of sirtuin 1 was assayed using TaqMan^®^ Gene Expression Assays (Thermo Fisher Scientific, Waltham, MA, USA) *SIRT1* (Hs1009006_m1), and Actin Beta (ACTB) (Hs1060665_g1) was chosen as endogenous control. All reactions were run on a 7900HT Fast Real-Time PCR System (Applied Biosystems, Foster City, CA, USA) in duplicates differing by <0.5 CT. miRNA reverse transcription was performed using a TaqMan miRNA Reverse Transcription Kit and specific primers (Thermo Fisher Scientific, Waltham, MA, USA) supplied with TaqMan microRNA Assays: *hsa-miR-181a* (Assay ID 000480, miRNA sequence: AACAUUCAACGCUGUCGGUGAGU), *hsa-miR-199a* (Assay ID 000498, miRNA sequence: CCCAGUGUUCAGACUACCUGUUC), and *RNU6B* (Assay ID 001093) as an endogenous control. RT reactions were performed according to the manufacturer’s instructions. All reactions were run in duplicates differing by <0.5 CT on 7900HT Fast Real-Time PCR System (Applied Biosystems, Foster City, CA, USA) in 96-well PCR plates. The data were analyzed using SDS 2.3 software and the relative expression of *SIRT1* and miRNA was calculated according to the ΔCt method. 

For the assessment of the SIRT1 intracellular protein level, the ELISA Human Sirtuin1 (SIRT1), catalog number SEE912Hu (Cloud-Clone Corp, Wuhan, Hubei, China) was used. The total protein level in the buffy coat samples was assessed with a BCA test—an average of 130 mg/mL. Based on the protein levels in the randomly selected samples from each of the tested groups, the ELISA test curve was determined 0.78–50 ng/mL. The tests carried out allowed us to draw the conclusion that the concentration of SIRT1 in the tested samples was below the detection level. Cell membrane and nuclear membranes lysis method optimization was applied. The selected methods included sonication on ice 4 times × 5 s with a break of 30 s, RIPA lysis buffer addition to the samples in three different layouts (1:1, 1:4, and 1:9—volume of sample: volume of lysis buffer), and a combination of sonication and buffer lysis. After thawing and membrane lysis, verification of cell membrane integrity by flow cytometry was performed. Enzyme-linked immunosorbent assay kits were used to assess HIF-1α (Invitrogen, Carlsbad, CA, USA) in the serum. The absorbance was measured at a λ = 450 nm wavelength by the absorbance reader (BioTek 800 TS, Agilent Technologies, Santa Clara, CA, USA).

Nominal data were presented as *n* with a percentage of the total; the proper tests were employed based on the size of the smallest subgroup. Given the non-normal distribution of the evening and morning concentrations of the examined microRNAs and *SIRT1* (Shapiro–Wilk test; all *p*-values < 0.05), nonparametric analyses were employed. All continuous variables were therefore presented as median with interquartile range (IQR: 1. quartile–3. quartile). Independent sample comparisons were conducted using the Mann–Whitney U test, whereas the Wilcoxon signed-rank test was applied for related samples to compare two groups. To examine the dependencies between more groups, a Kruskal–Wallis test with Dunn’s post hoc test were used. Correlations were assessed using the Spearman rank correlation coefficient. For evaluations involving more than two groups, the Kruskal–Wallis H test was utilized, followed by post hoc analyses to elucidate group differences. All statistical procedures were executed in STATISTICA version 13.PL (StatSoft, Tulsa, OK, USA).

## 3. Results

### 3.1. Study Groups Selection

There were 52 patients who met the inclusion criteria and were enrolled in the study, 4 of them were excluded due to PSG technical issues or withdrawal of consent for the morning blood collection. There were 17 (35%) in the control group, 11 (23%) severe OSA patients without T2DM, and 20 (42%) severe OSA patients with T2DM (see Table 1).

### 3.2. Evening and Morning Results Differences Within Groups

**Figure 3:**
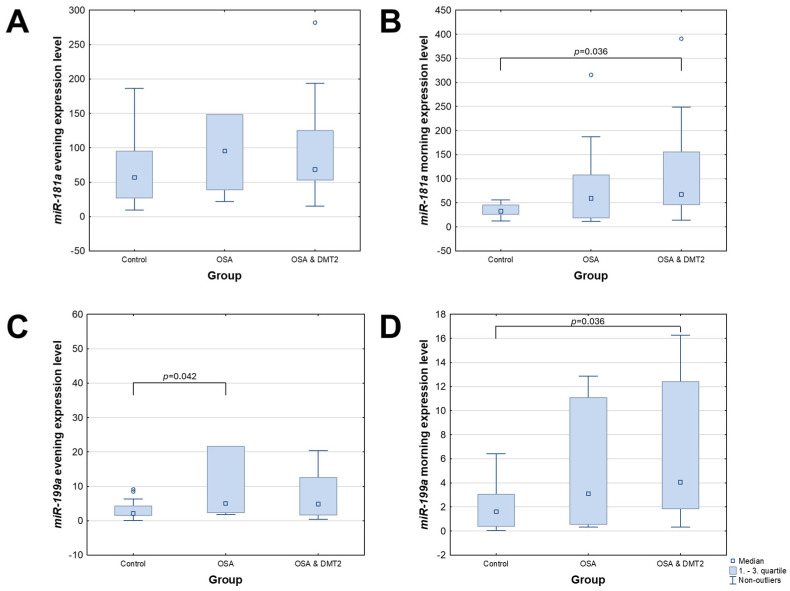
Dependencies in evening and morning expression levels of *miR-188a* and *miR-199a*. (**A**) evening expression of *miR-181a* (*p* = 0.191), (**B**) morning expression of *miR-181a* (*p* = 0.043), (**C**) evening expression of *miR-199a* (*p* = 0.038), (**D**) morning expression of *miR-199a* (*p* = 0.035). Results of post hoc (Dunn’s test) shown in figure. OSA—obstructive sleep apnea; T2DM—diabetes mellitus type 2.

We observed significant differences in the expression level of miRNA-199a in the evening (*p* = 0.038) and morning (*p* = 0.035; see Figure 3C,D) blood between all three groups. MiRNA-181a level showed significant differences only in the morning assessment (*p* = 0.043; see Figure 3A,B). Morning miRNA-181a expression was significantly higher in the OSA&T2DM group than in the control group: 67.618 (IQR: 47.502–152.966) vs. 32.685 (IQR: 25.883–45.448; *p* = 0.036; see Figure 3B). Evening miRNA-199a expression was significantly higher in the OSA group than in the control group: 5.043 (IQR: 2.445–16.989) vs. 2.081 (IQR: 1.520–4.276; *p* = 0.042; see Figure 3C). Its morning expression was significantly higher in the OSA&T2DM group than in the control group: 4.065 (IQR: 1.858–12.198) vs. 1.605 (IQR: 0.382–3.054; *p* = 0.036; see Figure 3D).

We did not observe similar dependencies for SIRT1 and HIF-1α plasma concentrations when assessing the blood samples taken in the evening and in the morning, which may be explained by the small sample size.

### 3.3. Alternative Group Formation and Statistical Analysis

Additionally, we have decided to perform an alternative group formation with statistical analysis. In this concept patients suffering from OSA were compared with patients without OSA and patients with T2DM were compared with patients not suffering from T2DM. A statistically significant difference in evening measurements between the groups of OSA patients and non-OSA patients was observed only in the case of miRNA-199a (5.043, IQR: 2.267–12.597 vs. 2.081, IQR: 1.52–4.946; *p* = 0.036). For morning measurements, both microRNAs were expressed differently: miRNA-181a (67.276, IQR: 40.099–139. 334 vs. 32.685, IQR: 25.883–45.448; *p* = 0.021) and miRNA-199a (3.102, IQR: 1.843–11.531 vs. 1.605, IQR: 0.382–3.054; *p* = 0.019; Table 2). Additionally, between the groups of patients with T2DM and the patients without T2DM, the statistically significant difference was found only for the morning expression level of miRNA-181a (67.618, IQR: 47.502–152.966 vs. 39.869, IQR: 25,464–66,761; *p* = 0.020; Table 3). The expression of the SIRT1 and HIF-1α serum protein levels did not differ between these groups.

### 3.4. SIRT1 Intracellular Protein Levels

Despite the damage to the cell and nuclear membranes, no intracellular SIRT1 protein was detected in any of the samples tested. Therefore, its intracellular concentration in the samples remained lower than the sensitivity of the enzyme immunoassay used thus the level of SIRT1 protein in all patient research groups was <0.31 ng/mL.

### 3.5. Correlations

**Figure 4:**
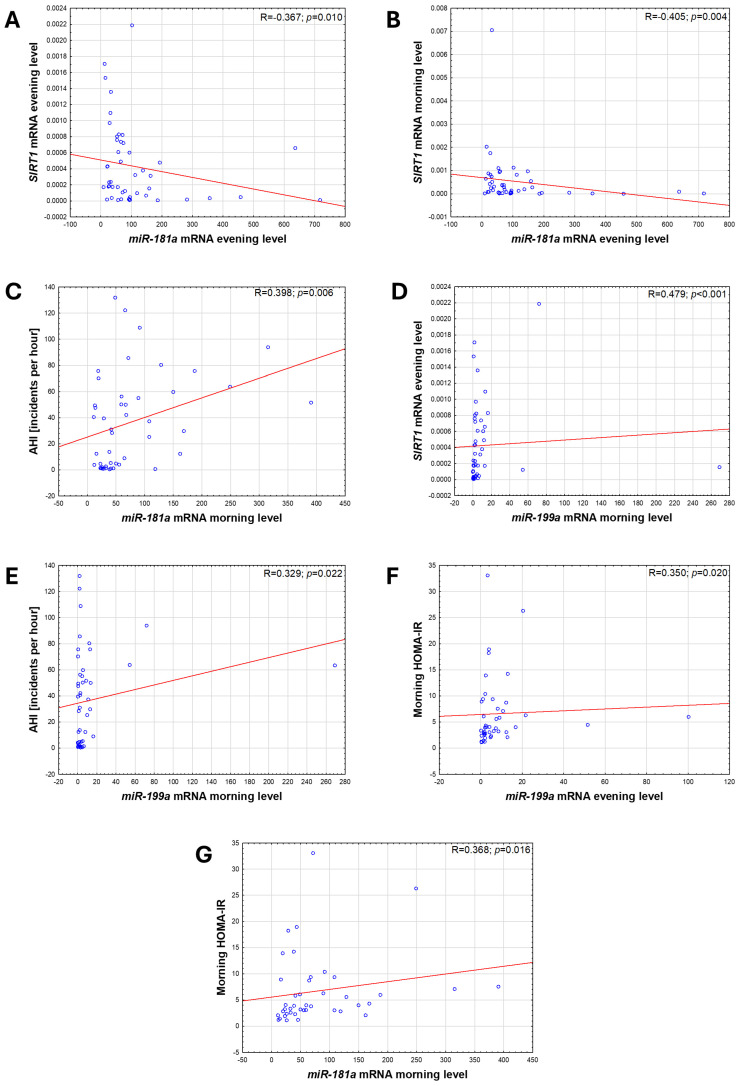
Correlations between expression levels of *miRNA-188a*, *miRNA-199a*, *SIRT1*, AHI, and HOMA-IR. (**A**) Evening expression of *miRNA-181a* and evening expression of *SIRT1*, (**B**) evening expression of *miRNA-181a* and morning expression of *SIRT1*, (**C**) morning expression of *miRNA-181a* and AHI, (**D**) morning expression of *miRNA-199a* and evening expression of *SIRT1*, (**E**) morning expression of *miRNA-199a* and AHI, (**F**) evening expression of *miRNA-199a* and HOMA-IR, (**G**) morning expression of *miRNA-181a* and HOMA-IR. Results of Spearman rank correlation shown in figure. SIRT1—sirtuin 1; AHI—apnea–hypopnea index; HOMA-IR—homeostasis model assessment-estimated insulin resistance.

The potential role of microRNAs in OSA and/or T2DM pathogenesis was indirectly assessed by correlations. MiRNA-181a evening expression presented a moderate negative correlation with the SIRT1 evening and morning expressions (R = −0.367, *p* = 0.010 and R = −0.405, *p* = 0.004, respectively). Moreover, morning miRNA-181a was positively corelated with AHI (R = 0.36, *p* = 0.012), HOMA-IR (R = 0.321, *p* = 0.034), and HIF-1α evening serum protein levels (R = 0.38, *p* = 0.01). MiRNA-199a evening expression presented a moderate positive correlation with the SIRT1 morning expressions (R = 0.48, *p* < 0.001) and HOMA-IR (R = 0.35, *p* = 0.02). While morning miRNA-199a was positively corelated with AHI (R = 0.33, *p* = 0.02) and HIF-1α evening serum protein levels (R = 0.32, *p* = 0.035). Only HOMA-IR (R = 0.58, *p* < 0.001) and morning miRNA-181a expression levels were significantly correlated with BMI (R = 0.31, *p* = 0.03). For all other variables, correlations with BMI remained not significant. There was no statistically significant correlation between SIRT1 and HIF-1α serum protein levels and other variables. All the correlations are depicted in Figure 4.

## 4. Discussion

### 4.1. Hypoxia-Sensitive microRNA

In our study, the expression of both *miRNA-199a* and *miRNA-181a-1* was found to be increased in the patients suffering from OSA compared to the healthy controls. These observations are not consistent with the Santamaria-Marios et al. study. The validated qPCR expression of morning *miRNA-199a* in the study of Santamaria-Marios et al. was decreased in the OSA patients: a 0.54-fold change in the morning (*p* = 0.184). In our study, we observed a 2.4-fold increase in the evening (*p* = 0.036) and a 1.9-fold increase in the morning (*p* = 0.018). The differences for *miRNA-181a* between the study of Santamaria-Marios et al. and our study were also present. The morning expression of *miRNA-181a* in the study of Santamaria-Marios et al. was found to be lower in the OSA patients, a 0.59-fold decrease (*p* = 0.001) compared to a 2.1-fold increase (*p* = 0.021) in our study. The described differences may result from the selection of the tested material—in our study we used buffy coat, while in the study of Santamaria-Marios et al., plasma was used [25]. Since both transcription and translation occur intracellularly, the assessment of the intracellular expression levels is an important element to fully understand the described molecular mechanisms. A comparison of the results from both studies may suggest that *miRNA-181a* and *miRNA-199a* are vulnerable to any contamination of samples with blood cells or cell lysis because they present an opposite reaction to hypoxic conditions depending on the material—blood cells or plasma. Any contamination of plasma with blood cells can lead to false results. This may suggest a reduced usefulness of both miRNAs as biomarkers for OSA diagnosis. The contamination of blood cells with plasma components may also affect the expression results however this type of impurity is of lower clinical significance. The negative correlation between the expression of *SIRT1* and *miRNA-181a* described in our study is consistent with the findings of Zhou et al.’s study, which was performed on cell cultures [32]. A decreased expression of *miRNA-181a* leads to *SIRT1* overexpression, which has a beneficial effect on glucose metabolism [19,33], and in the OSA patients, we observed the opposite. Both the increased *miRNA-181a* expression in the OSA patients and the negative correlation between *miRNA-181a* and *SIRT1* may suggest that one of the causes of the increased incidence of T2DM in the OSA patients is the epigenetic mechanism based on the above described relationship.

### 4.2. Concentration of SIRT1 Protein in Blood Cells from OSA Patients

In the study of Chen et al., the level of SIRT1 protein level in peripheral blood mononuclear cells was assessed, and the average concentration of SIRT1 protein in the OSA patients was found to be 0.55 pg/μg of total protein. Additionally, the detection limit of the ELISA assay was 30 pg/mL. In our study, the detection level of SIRT1 protein was 0.31 ng/mL, which when related to total protein concentration (130 mg/mL) gives 0.002 pg/μg [16]. However, due to the lack of information on the total protein concentration in Chen’s study, the test results cannot be directly compared with each other. Encouraged by the results of Chen et al., we attempted to assess the concentration of the SIRT1 protein in blood cells. Our results remained consistent with the information from the Protein Human Atlas www.proteinatlas.org (accessed on 15 January 2024), where no expression of the SIRT1 protein was found in either blood cells or plasma.

### 4.3. Limitations

Increased BMI and obesity are the main risk factors for both OSA and T2DM development. Due to this, the selection of a control group is challenging. Even though we tried to select patients with the most similar BMI between the groups, the differences in mean BMI score were up to 6 kg/m^2^ between the groups. This is the main limitation of our study. On the other hand, all patients included in the study were obese and exposed to obesity consequences. The majority of patients in the control and OSA groups have class 1 obesity, and the majority of patients from the group OSA&T2DM have class II obesity. Furthermore, it was proven that OSA is a risk factor for T2DM development independently of obesity and other factors [34,35]. In our study, we found only one statistically significant correlation between analyzed factors and BMI. It was a weak correlation between morning *miRNA-181a* expression levels and BMI; correlations with other variables remained insignificant. Other limitations of our study include the small number of participants and limited clinical significance of found correlations [36]. Additionally, possible contamination of the buffy coat with plasma components and low levels of *SIRT1* expression may have occurred.

## 5. Conclusions

Patients suffering from OSA and T2DM had an increased expression of *miRNA-181a*. Moreover, a negative correlation between *miRNA-181a* and *SIRT1* expression was observed, while a correlation between *miRNA-181a* and insulin resistance was positive. This phenomenon might suggest a possible epigenetic pathway for the increased incidence of T2DM in OSA patients. This study is burdened with numerous limitations; thus, to confirm the role of *miRNA-181a*, further investigation and larger groups of patients are necessary. Additionally, *miRNA-181a* and *miRNA-199a* may be sensitive to any cellular contamination, reducing their chances of being implemented in clinical use for OSA diagnosis as serum biomarkers.

Further research on the relationship between the selected miRNAs, SIRT1, and HIF-1α in patients suffering from OSA may lead to a better understanding of the mechanisms in response to hypoxia and its complications. Furthermore, this may help to determine novel diagnostic methods, predict OSA phenotype, and indicate possible future therapeutic targets for OSA patients.

## Figures and Tables

**Table 1:** Demographic and clinical characteristics of study and control groups. OSA—obstructive sleep apnea; T2DM—diabetes mellitus type 2; AHI—apnea–hypopnea index; BMI—body mass index.

	Control Group	OSA Group	OSA&T2DM Group
*n*	17 (35%)	11 (23%)	20 (42%)
Sex [male]	13 (76%)	9 (81%)	15 (75%)
Age [years old]	47 (IQR: 44–60)	48 (IQR: 43–63.5)	57.5 (49–64.5)
BMI [kg/m^2^]	30.450 (IQR: 27.7–31.8)	34.602 (IQR: 30.6–37.2)	36.977 (IQR: 32.2–41.6)
AHI [incidents/h]	1.5 (IQR: 1.0–3.9)	50.2 (IQR: 40.0–75.7)	50.9 (IQR: 27.6–65.4)

**Table 2:** Baseline characteristics and results of *miRNA-181a*, *miRNA-199a*, and *SIRT1* in subgroups OSA (OSA + OSA and T2DM group) vs. no OSA (controls). ♀—female. OSA—obstructive sleep apnea; T2DM—diabetes mellitus type 2; BMI—body mass index; *SIRT1*—sirtuin 1.

	No OSA *n* = 17	OSA *n* = 31	*p*-Value
Sex	23.5% ♀	22.6% ♀	-
Age	49.9 ± 8.1	55.1 ± 11.9	-
BMI [kg/m^2^]	30.24 ± 4.74	35.65 ± 6.31	-
*miRNA-181a* evening	56.946 (27.03–94.88)	79.188 (53.34–125.18)	0.07
*miRNA-199a* evening	2.081 (1.52–4.95)	5.043 (2.27–12.60)	0.036
*SIRT1* evening	1.83 × 10^−4^ (2 × 10^−5^–8.22 × 10^−4^)	3.12 × 10^−4^ (8.8 × 10^−5^–6.88 × 10^−4^)	>0.05
*miRNA-181a* morning	32.685 (25.88–45.45)	67.276 (40.10–139.33)	0.021
*miRNA-199a* morning	1.605 (0.38–3.05)	3.102 (1.84–11.53)	0.018
*SIRT1* morning	2.39 × 10^−4^ (2.6 × 10^−5^–6.52 × 10^−4^)	1.9 × 10^−4^ (0.66 × 10^−5^–6.47 × 10^−4^)	>0.05

**Table 3:** Baseline characteristics and results of *miRNA-181a*, *miRNA-199a*, and *SIRT1* in subgroups T2DM vs. no T2DM (OSA + controls). ♀—female. OSA—obstructive sleep apnea; T2DM—diabetes mellitus type 2; BMI—body mass index; SIRT1—sirtuin 1.

	No T2DM *n* = 28	T2DM *n* = 20	*p*-Value
Sex	21.4% ♀	25% ♀	-
Age	50.9 ± 10.3	56.5 ± 11.1	-
BMI [kg/m^2^]	31.64 ± 5.16	36.67 ± 6.73	-
*miRNA-181a* evening	69.018 (28.54–97.05)	68.441 (53.36–119.01)	>0.05
*miRNA-199a* evening	4.018 (1.94–8.63)	4.838 (1.80–12.37)	>0.05
*SIRT1* evening	1.79 × 10^−4^ (2 × 10^−5^–6.21 × 10^−4^)	3.51 × 10^−4^ (1.47 × 10^−4^–7.41 × 10^−4^)	>0.05
*miRNA-181a* morning	39.869 (25.46–66.76)	67.618 (47.50–152.97)	0.019
*miRNA-199a* morning	2.173 (0.43–4.75)	4.065 (1.86–12.20)	0.07
*SIRT1* morning	1.34 × 10^−4^ (3 × 10^−5^–6.94 × 10^−4^)	3.23 × 10^−4^ (6.7 × 10^−5^–6 × 10^−4^)	>0.05

## Data Availability

The original data presented in the study are openly available in RepOD—at https://doi.org/10.18150/ATJL3W.
